# Bone *FGF23* expression and renal regulatory responses to excess phosphate consumption by juvenile swine

**DOI:** 10.1093/jbmrpl/ziag129

**Published:** 2026-08-08

**Authors:** Mariola Grez-Capdeville, Brittney P Kokinos, Laura A Amundson, Thomas D Crenshaw

**Affiliations:** Department of Animal and Dairy Sciences, University of Wisconsin-Madison, Madison, WI 53706, United States; Department of Animal and Dairy Sciences, University of Wisconsin-Madison, Madison, WI 53706, United States; Department of Animal and Dairy Sciences, University of Wisconsin-Madison, Madison, WI 53706, United States; Department of Animal and Dairy Sciences, University of Wisconsin-Madison, Madison, WI 53706, United States

**Keywords:** phosphatonins, kidney, bone, homeostasis, mineral nutrition, porcine model

## Abstract

Fibroblast growth factor 23 (FGF23), a bone-derived hormone, regulates phosphorus (P) homeostasis by modulation of renal P excretion and vitamin D metabolism. Physiological roles of FGF23 have been mainly characterized in human disorders of P metabolism and murine models. This study aimed to validate roles of FGF23 in P homeostasis in a large animal, healthy swine model. Juvenile pigs fed a low-P diet (LP) for 4 d were fasted overnight then fed a high-P diet (HP) for 5 d. Blood was collected to determine plasma concentrations of P, calcium (Ca), PTH, and vitamin D metabolites, and urine was collected to assess P and Ca concentrations. Femur and kidney tissues were collected to assess gene and protein expression related to FGF23 synthesis, signaling, and vitamin D metabolism. The rapid increase in plasma P (5 to 18 mg/dL) within 12 h of HP intake was lagged by increased urinary P. At maximal urinary P concentration (108 h), plasma P had returned to physiological ranges. The initial increase in plasma PTH with HP intake returned to baseline levels by 108 h. At 108 h, HP consumption upregulated bone *FGF23* mRNA expression (250-fold increase relative to LP). Sodium-phosphate co-transporters (*NaPi2a* and *NaPi2c*) mRNA and NaPi2a protein expression decreased with HP intake, consistent with FGF23-mediated phosphaturia, independent of circulating PTH. Bone mRNA expression of post-translational FGF23 regulators (*GALNT3* and *FURIN*) was unaffected by HP consumption, whereas kidney FGF23 receptors (*FGFR1* and *αKLOTHO*) slightly increased. High P intake downregulated renal 1α-hydroxylase (*CYP27B1*) mRNA expression at 108 h, consistent with decreased circulating 1,25(OH)_2_D_3_ concentrations. In conclusion, HP consumption stimulated bone *FGF23* expression and renal adaptive responses in healthy pigs, consistent with roles of FGF23-mediated phosphaturia and regulation of vitamin D metabolism for maintenance of P homeostasis, as previously described in humans and rodents.

## Graphical Abstract

**Graphical Abstract:**
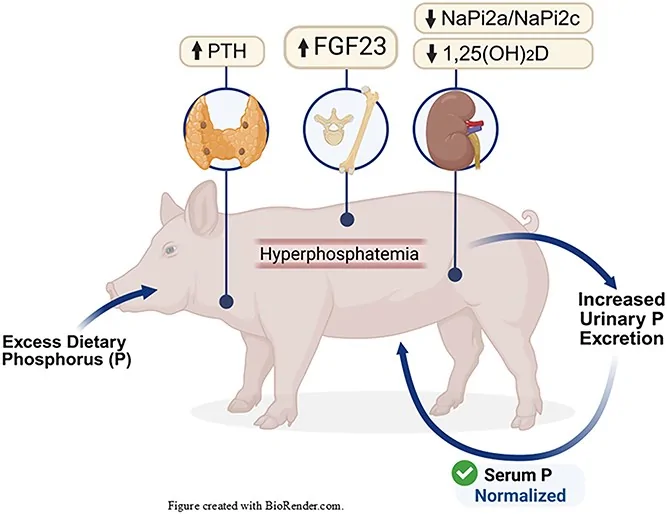

## Introduction

Phosphorus, as ortho-phosphate (P), is an essential macromineral involved in multiple biological processes and is one of the major structural components of the bone mineral matrix. Fibro-blast growth factor 23 (FGF23) is a bone-derived protein hormone that contributes a central role in the regulation of P homeostasis in the bone-renal axis. The primary physiological function of FGF23 for maintenance of normophosphatemia is to reduce renal P reabsorption in response to excessive P loading, thereby increasing urinary P excretion and promoting the restoration of normal circulating P concentrations. In addition to phosphotropic effects, FGF23 also regulates vitamin D metabolism, a key hormone involved in mineral metabolism and skeletal health.^1^^,^^2^

Fibrob-last growth factor 23 is predominantly produced by osteocytes and osteoblasts in bone.^3^ The synthesis of FGF23 is regulated at both transcriptional and post-translational levels by multiple factors, including dietary P intake, hyperphosphatemia, circulating PTH concentrations, and the active metabolite of vitamin D, 1,25(OH)_2_D_3_.^4–6^ Post-translational modifications also occur within bone cells to control the proteolytic cleavage of FGF23. In particular, O-glycosylation protects FGF23 from cleavage and is therefore required for the secretion of biologically active, full-length FGF23.^7–9^ After secretion into circulation, full-length FGF23 exerts canonical signaling in target organs through interactions with FGF receptor 1 (FGFR1) and the co-receptor αKlotho. This co-receptor converts the ubiquitously expressed FGFR1 into a specific receptor for FGF23^10^ and is predominantly expressed in kidneys and parathyroid glands.^11^

Kidneys are the main organs involved in maintaining P homeostasis through adjustments in the amount of P reasorbed or excreted via urine. In kidneys, the interaction of FGF23 with the FGFR1-αKlotho receptor complex results in the reduction of renal P reabsorption by decreased expression and abundance of renal sodium-phosphate cotransporters of the SLC34 family (NaPi2a and NaPi2c) and the SLC20 family (PiT2).^12^^,^^13^ In addition to phosphaturic action, FGF23 reduces the expression of renal 1α-hydroxylase (*Cyp27b1*), the enzyme required for the synthesis of 1,25(OH)_2_D_3_, thereby indirectly decreasing intestinal P absorption.^14^ Therefore, under healthy conditions, the overall effects of FGF23 result in reduced renal P reabsorption and intestinal P absorption, maintaining normophosphatemia.

Current knowledge of the biological roles of FGF23 is largely based on studies conducted in human subjects with renal failure or genetic disorders of P metabolism, as well as murine models. Swine, including both domestic and miniature pigs, are extensively studied as large animal models in biomedical research.^15–17^ The anatomical and physiological similarities between swine and humans provide significant advantages over other animal models. In terms of skeletal health, pigs share many similarities with humans in the regulation of Ca, P, and vitamin D metabolism.^18^^,^^19^ Pigs have been used to study various skeletal disorders and diseases, including kyphosis, rickets, osteoporosis, and metabolic bone disease of prematurity.^20–23^ Furthermore, the size of pigs makes them a suitable model for surgical procedures and bone fracture healing studies, allowing for the use of human-sized implants and instruments, and enhancing the translational potential of preclinical studies. Since alterations in mineral metabolism can negatively impact bone health, studying FGF23 as a regulator of P homeostasis in swine models will expand our understanding of P metabolism in swine and improve the biocompatibility knowledge between swine and humans. In this study, we aimed to validate the roles of FGF23 in healthy pigs by analyzing changes in biological molecules that modulate the bone-kidney endocrine axis and physiological adaptations driven by high P intake.

## Materials and methods

All animal procedures were approved by the University of Wisconsin-Madison IACUC (protocol A006387). Pigs were housed at the University of Wisconsin-Madison Livestock Laboratory facility throughout the study.

### Animals and dietary treatments

**Figure 1:**
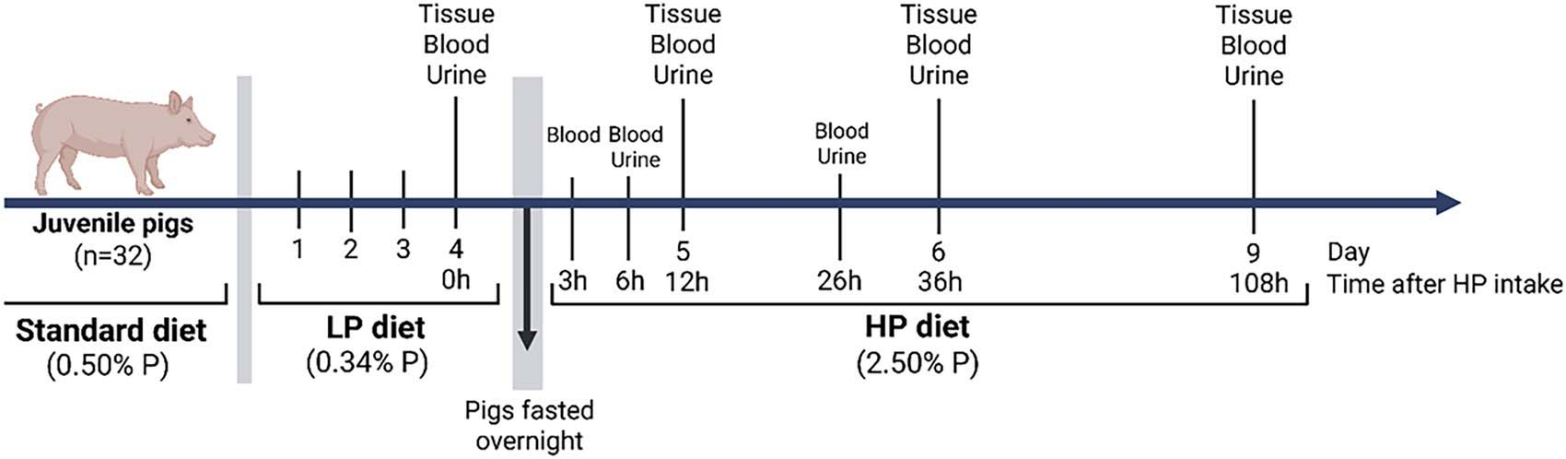
Experiment design and timeline. Figure created with BioRender.com.

Thirty-two crossbred barrows were used in duplicate trial blocks (*n* = 16/block) with staggered start dates to facilitate collections. The average initial BW was 24.5 kg (ranging from 21.1 to 27.5 kg). Pigs were housed in individual pens for a 9-d study period. Two experimental diets (Table 1) were formulated to provide either marginal (low-P [LP]; 0.34% P) or excessive (high-P [HP]; 2.50% P) P concentrations. All pigs were fed the LP diet for 4 d. At the end of day 4, pigs were fasted overnight and then transitioned to the HP diet the following morning for 5 d. Other than the overnight fasting period, pigs were allowed continuous access to feed and water throughout the experiment. Randomly selected subsets of pigs (*n* = 8 per time) were euthanized by electrical stunning and exsanguination for tissue sample collections at the end of the LP feeding period (baseline; 0 h), and at 12, 36, and 108 h intervals after HP feeding. Feed intake and BW weights were recorded prior to euthanasia. Blood and spot urine samples were collected at designated intervals during the experiment as illustrated in Figure 1.

**Table 1:** Feed ingredients and analyzed nutrient composition of the low (LP) and high-phosphorus (HP) diets (as-fed basis) fed to juvenile pigs.^a^

**Item**	**Units**	**LP**	**HP**
**Ingredients**			
**Corn**	g/kg	753.7	623.5
**Soybean Meal, solv. extract**	g/kg	204.6	215.6
**L-lysine mHCl**	g/kg	2.0	2.0
**Threonine**	g/kg	0.5	0.5
**Monocalcium phosphate**	g/kg	0.0	101.6
**Calcium carbonate**	g/kg	5.7	23.3
**Animal-vegetable blend fat**^**b**^	g/kg	20.0	20.0
**Sodium chloride**	g/kg	3.5	3.5
**Vitamin-mineral premix**^**c**^	g/kg	10.0	10.0
**Calculated nutrient content**			
**Metabolizable energy**	kcal/kg	3397	2992
**Crude protein**	g/kg	162.0	157.0
**Lysine**	g/kg	9.5	9.5
**Calcium**	g/kg	3.8	27.7
**Total phosphorus**	g/kg	3.4	25.0
**Ca to total P ratio**	-	1.11	1.11
**Analyzed nutrient content**			
**Calcium**	g/kg	4.1	25.3
**Total phosphorus**	g/kg	3.2	24.9
**Ca to total P ratio**	-	1.28	1.02

a All pigs (*n* = 32) were fed LP diet for 4 d, fasted overnight, and then fed HP diet for 108 h.

b Blend of animal and vegetable fats (Maxco Inc.).

c The vitamin-mineral premix provided the following nutrients per kilogram of diet: vitamin A, 2800 IU; vitamin D, 280 IU; vitamin E, 14 IU; vitamin K, 0.75 IU; niacin, 22 mg; pantothenic acid, 12 mg; riboflavin, 8 mg; vitamin B_12_, 33 μg; copper, 1.5 mg; iodine, 0.3 mg; iron, 38 mg; selenium, 0.2 mg; and zinc, 90 mg.

### Blood, urine, and tissue collections

Blood samples were collected into EDTA tubes (10 mL) by venipuncture. Samples were centrifuged at 1500 × g for 12 min, and plasma aliquots were stored at −80° C for subsequent analyses. Free-catch urine samples were collected into plastic bags strapped around the body, as previously described.^24^ After urination, samples were transferred into sterile containers, and aliquots were stored for mineral (30 mL, 4° C) and creatinine (2 mL, −20° C) analyses. Kidney cortex and bone tissue samples were collected immediately after euthanasia. Cortical bone samples were taken from the midshaft of the right femur. All tissue samples were snap-frozen in liquid nitrogen immediately after collection and stored at −80° C until use.

### Plasma, urine, and feed analyses

Plasma PTH concentrations were measured using the MicroVue Human Bioactive PTH 1-84 EIA kit (cat #60-3000; Quidel Corporation). Concentrations were based on duplicate analysis for all samples, and the calculated inter- and intra-assay coefficients of variation were 3.9% and 5.4%, respectively. Plasma concentrations of vitamin D metabolites (25(OH)D_3_, 24,25(OH)_2_D_3_, and 1,25(OH)_2_D_3_) were analyzed by LC-MS/MS at Heartland Assays, Inc. Urinary creatinine concentrations were determined using Jaffe’s colorimetric method. Concentrations of P and Ca in plasma, urine, and feed samples were determined following procedures previously described.^25^ Briefly, urine and feed samples were digested in a nitric-perchloric acid mixture. Concentrations of Ca in plasma, urine digests, and feed digests were determined by flame atomic absorption spectrometry (iCE 3000 Series AA Spectrometer, Thermo Fisher Scientific). Phosphorus concentrations in the feed and urine digests were quantified by a colorimetric molybdovanadate method and plasma P by molybdenum blue method using spectrophotometry (Gilford Spectrophotometer 260, Gilford Instrument Laboratories, Inc.).

### Renal brush border membrane vesicles preparation and protein isolation

Renal brush border membrane vesicles (BBMv) were isolated from kidney cortex samples using the Mg precipitation method previously described.^26^ Briefly, kidney cortex tissue (1 g) was homogenized in mannitol-Tris buffer (10 mL), pH 7.1. The homogenate was centrifuged at 200 × *g* for 2 min to remove unbroken tissue and large debris. A homogenate aliquot (300 μL) was mixed with radioimmunoprecipitation assay (RIPA) lysis and extraction buffer (600 μL), incubated on a rocker at 4° C for 30 min, centrifuged at 13 000 × *g* for 20 min at 4° C, and the supernatant was saved for protein analysis of the whole tissue lysate. The remaining homogenate was subjected to 2 consecutive precipitations with MgCl_2_ in mannitol-Tris buffer (12 mM) to obtain a pellet of microvillus membrane vesicles. The BBMv pellet was resuspended in RIPA buffer containing protease inhibitors and stored at −80° C. Protein concentration was quantified using the bicinchoninic acid protein assay. The extracted proteins were stored at −80° C for Western blot analysis.

### Western blotting

Total kidney cortex homogenate (25 μg) and BBMv samples (35 μg) were diluted with Laemmli sample buffer and loaded on 10% sodium dodecyl sulfate-polyacrylamide gel. Proteins were separated by electrophoresis (70 V for 30 min, 120 V for 90 min) and transferred to polyvinylidene fluoride membranes (200 mA for 90 min). After transfer, the membranes were blocked with 5% non-fat dry milk in Tris-buffered saline containing 0.1% Tween 20 (TBST) and then incubated in the primary antibody overnight at 4° C. The following primary antibodies were used: anti-Klotho (1:1000; PA5-99961, Invitrogen), anti**-**SLC34A1 (1:1000; PA5-113005, Invitrogen), anti**-**SLC34A3 (1:1000; NBP2-93939, Novus Biologicals), and anti-β-actin (1:2000; sc-47778, Santa Cruz Biotechnology). Membranes were washed three times (10 min each) in TBST and incubated with the secondary antibodies anti-rabbit IgG horseradish peroxidase (HRP)-linked (0.5:1000, 7074S, Cell Signaling Technology) or anti-mouse HRP-linked (0.5:1000; sc-2314, Santa Cruz Biotechnology). The signal was visualized using the C-Digit Blot scanner after incubation in chemiluminescence substrate. Densitometry analysis and quantification were performed using ImageJ software.

### Total RNA purification and quantitative RT-PCR

The mRNA expression of *FGF23* was analyzed in tissue samples collected at 0, 12, 36, and 108 h after HP. The expression of *SOST*, which is expressed primarily by osteocytes, the bone cells that produce FGF23, along with genes associated with the regulation of post-transcriptional cleavage of FGF23 (*GALNT3*, *FURIN*, and *FAM20c*), FGF23 renal receptors (*FGFR1* and *αKLOTHO*), and vitamin D metabolism (*Cyp27b1* and *CYP24A1*) were assessed. Cortical bone samples were pulverized in liquid nitrogen. Bone powder and kidney cortex samples were homogenized in TRI-Reagent (Molecular Research Center) with a tissue grinder. The homogenized tissue was centrifuged for 5 min at 12 000 × *g* at 4° C to remove particulate debris, and the supernatant was used for total RNA isolation with the Direct-zol RNA MiniPrep kit (R2050; Zymo Research). Total RNA was reverse transcribed to cDNA using the high-capacity cDNA Reverse Transcription Kit (4368814; Applied Biosystems). Amplification was performed on a Bio-Rad CFX96 system using the SsoFast EvaGreen Supermix and the following conditions: enzyme activation at 95° C for 30 s, followed by 40 cycles of a 2-step reaction denaturation at 95° C for 5 s, and annealing/extension at 60° C for 20 s. A melting curve analysis was performed after the amplification cycles to assess single-product amplification. All reactions were run in duplicates, and controls were included on each plate. Primer sequences are provided in Table S1. The mRNA expression of target genes was normalized to the expression of housekeeping genes (*RPS15*, *RPL13A*, and *ACTB*), and relative expressions to the control (LP) were calculated using the 2^-ΔΔC_T_^ method.

### Statistical analysis

Data were analyzed using SAS version 9.2 software (SAS Institute Inc.) or GraphPad Prism software. Normal distribution of the data was tested by Shapiro–Wilk normality test. Descriptive statistics for feed intake, P intake, and body weights are presented as mean *+* SEM. Statistical differences for plasma and urine variables were analyzed using repeated measurements ANOVA test followed by multiple comparisons against the control treatment (LP, 0 h) with Dunnet’s adjustment. Time and trial blocks were used as fixed effects, and the interaction of time and trial block as a random effect. Real-time qPCR data did not fulfill the normality assumption. Therefore, qPCR data were analyzed by non-parametric Kruskal–Wallis test, followed by Dunnett’s test for multiple comparisons against the control (LP). The level of significance was set as *p* < .05.

## Results

### Body weight and P intake

Based on observations of growth, feed consumption and behavioral traits, all animals were healthy throughout experiment. Pigs gained an average of 2.5 kg over the 5-d HP feeding period, which is adequate for pigs of this age. Differences in feed intake were not detected between the dietary P concentrations (Table 2). Total P intake over the 4 d of LP diet was comparable to the amount of P consumed during the first 12 h of HP feeding (21 *+* 1 vs 23 *+* 2 g), resulting in an acute increase in dietary P load that triggered physiological adaptations to maintain P homeostasis.

**Table 2:** Descriptive statistics for body weights, feed intake, and P intake during the experiment.

		**Time after HP intake, h** ^**a**^			
**Item**	**Units**	**0**	**12**	**36**	**108**
**Pig weight**	kg	27.5 *+* 0.3	−	27.4 *+* 0.8	30.0 *+* 1.0
**Feed intake**	g/h	73 *+* 3	75 *+* 7	60 *+* 2	68 *+* 4
**−**	kg/period^b^	6.1 *+* 0.2	0.9 *+* 0.1	2.1 *+* 0.1	7.3 *+* 0.4
**P intake**	g/h	0.25 *+* 0.01	1.88 *+* 0.16	1.49 *+* 0.04	1.69 *+* 0.10
**−**	kg/period^b^	21 *+* 1	23 *+* 2	54 *+* 2	183 *+* 11

a Pigs were fed a low P diet for 4 d, fasted overnight, and then fed a high P (HP) diet. Data are means *+* SEM for 8 pigs euthanized for tissue collection at 0 h (pre-fasting) and 12, 36, and 108 h post-fast.

b Cumulative feed and P intake at 0 (4 d pre-fast) and 12, 36, and 108 h (post-fasting).

### Plasma concentrations and urinary excretion of P and Ca

**Figure 2:**
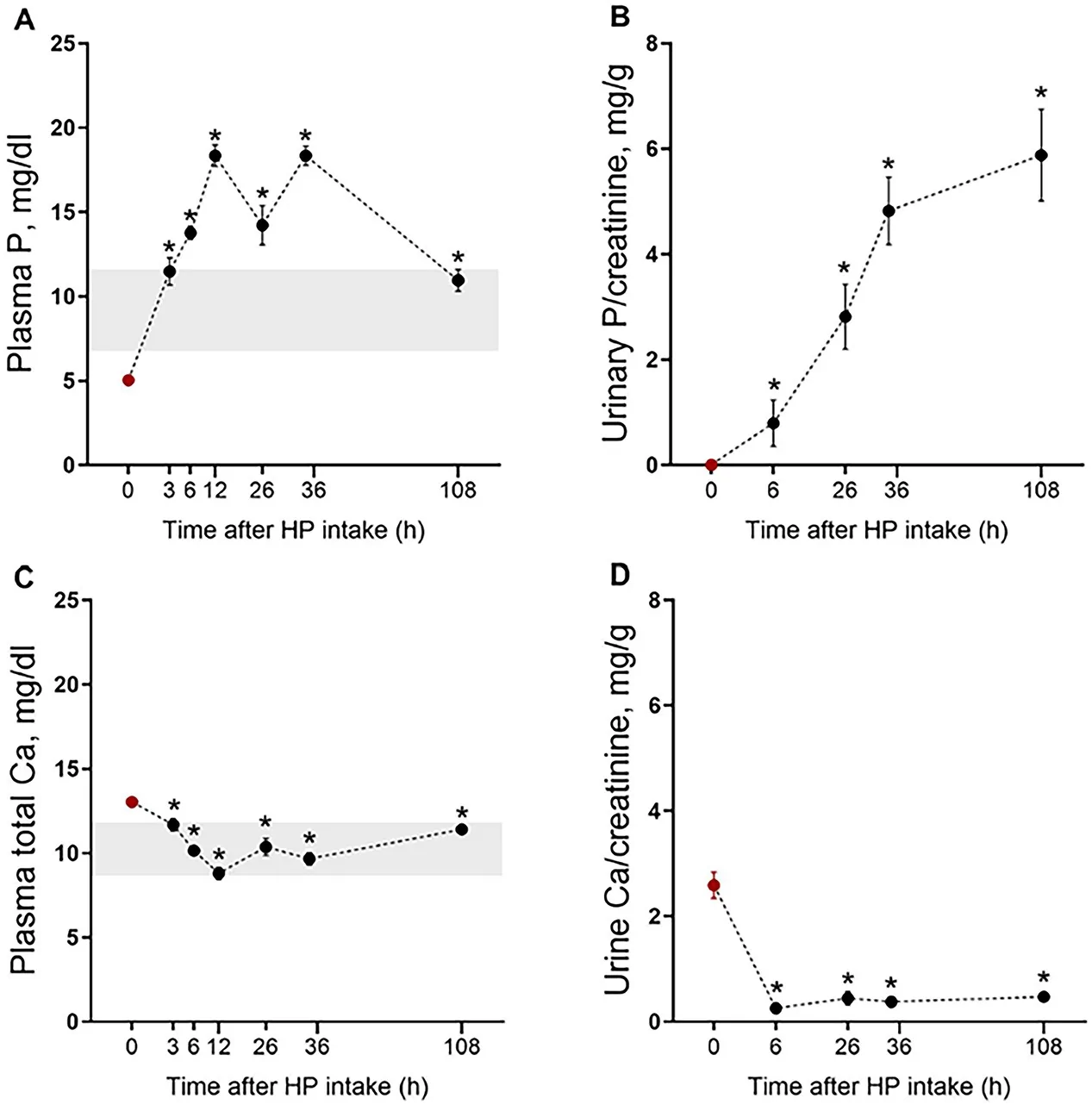
Changes in plasma (A) and urine (B) concentrations of P and Ca (C and D) following high phosphorus (HP) intake. Shaded areas represent physiological ranges for P (7-11.5 mg/dL) and Ca (8.5-11.5 mg/dL) plasma concentrations. Data are presented as mean *+* SEM, *n >* 8 pigs per group. ^*^Difference from 0 h, *p* < .05.

Plasma P concentration rapidly increased within the first 3 h following the increase in dietary P and continued to increase over the first 12 h before apparently plateauing between 12 and 36 h (Figure 2A). Plasma P concentrations returned to normal physiological values by 108 h after the start of HP feeding. Urinary P concentration (Figure 2B) lagged the increase in plasma P concentrations. The initial, rapid increase in urine P excretion was maintained through the first 36 h with high concentrations still evident at 108 h, even after plasma P concentrations were restored to physiological ranges. Less dramatic differences were noted in plasma and urinary Ca concentrations. Both plasma and urinary Ca concentrations were higher during the consumption of the LP diet (Figure 2C and D). However, within 6 h of HP consumption, plasma Ca returned to normal ranges, and urinary Ca concentration decreased and remained low and constant until the end of the experiment. These findings indicate renal physiological adaptations that maintain P and Ca homeostasis.

### Plasma PTH and vitamin D metabolites

**Figure 3:**
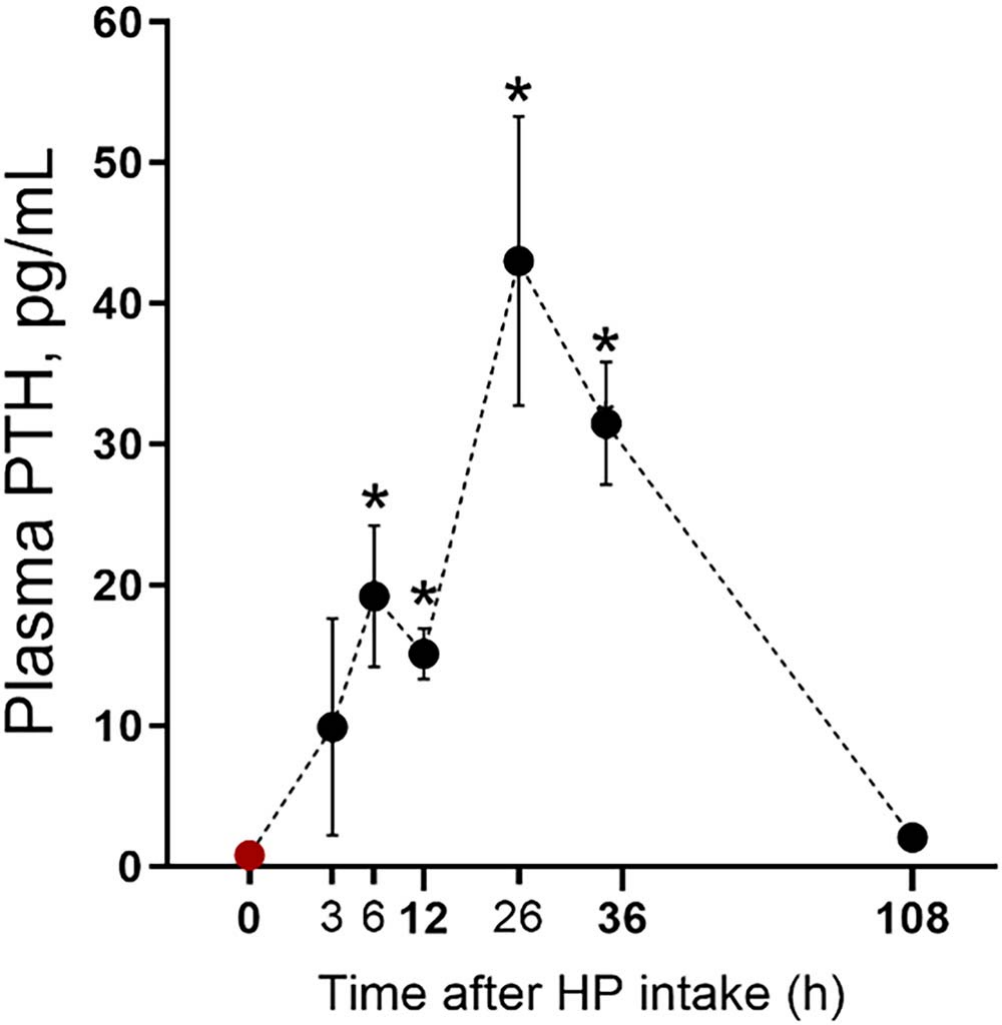
Effects of high phosphorus (HP) intake on plasma concentrations of PTH. Data are presented as mean *+* SEM, *n* = 5-8 pigs per group. ^*^Indicates difference from 0 h, *p* < .05.

Plasma PTH concentrations increased following HP intake (Figure 3), peaking at 26 h and returning to baseline values by 108 h of HP feeding. Interestingly, urinary P concentrations remained elevated at 108 h, inferring phosphaturia effects that were independent of PTH. Plasma 25(OH)D_3_ concentrations did not change over the 108 h of HP feeding (Table 3). Plasma 24,25(OH)_2_D_3_ was detected in only 25% of samples analyzed. However, both the concentration and the number of pigs with detectable levels were higher at 108 h after HP intake compared to baseline. In contrast, plasma 1,25(OH)_2_D_3_ concentrations decreased significantly over 108 h after pigs consumed the HP diet.

**Table 3:** Effects of high phosphorus (HP) diet on plasma concentrations of vitamin D metabolites.

		**Time after HP intake, h**				
**Metabolite** ^**a**^	**Units**	**0**	**12**	**108**	**SEM**	***p***
**25(OH)D**_**3**_	ng/mL	5.1	4.6	4.7	0.6	.693
**24,25(OH)**_**2**_ **D**_**3**_ ^**b**^	ng/mL	0.3	−	2.1	−	−
**1,25(OH)**_**2**_ **D**_**3**_ ^**c**^	pg/mL	244^x^	210^x^	46^y^	20	<.001

a Values are means for each metabolite with a concentration above the detection limit for the respective metabolite. The total number of samples analyzed was 8 pigs per group. For the 24,25(OH)_2_D_3_ values only 1, 0, or 5 samples were above detection limits at 0, 12, and 108 h, respectively.

b Statistical analysis was not performed due to low sample numbers with detectable concentrations of 24,25(OH)_2_D_3_.

c Means within a row with different superscript letters (x and y) differ, *p* < .05.

### Changes in gene mRNA and protein expression in bone and kidney

**Figure 4:**
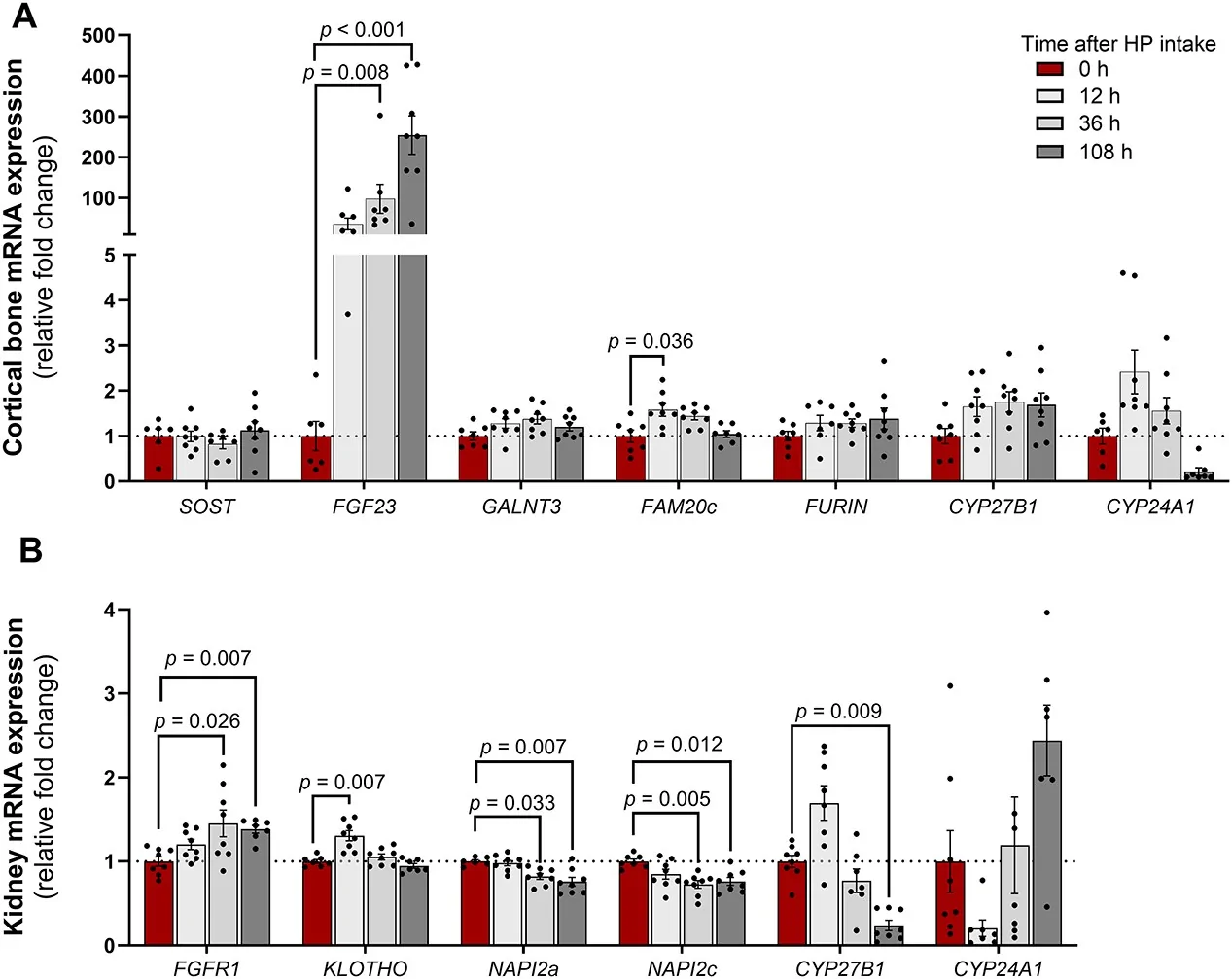
Relative expression (RT-qPCR) of genes involved in FGF23 synthesis and signaling pathways and P metabolism in (A) femoral cortical bone and (B) kidney cortex samples. Target gene expression was normalized to the geometric mean of 2 housekeeping genes (bone: *RPS15A* and *RPL13A*; kidney*: RPS15A* and *ACTB*), and fold-change was calculated relative to the 0 h time point. Data are presented as mean *+* SEM; *n* = 7-8 pigs per group. A *p*-value <.05 was considered significant.

In cortical bone (Figure 4A), *SOST* mRNA levels were not affected by dietary P. *FGF23* mRNA expression was markedly increased by the HP diet, with approximately a 35-fold increase at 12 h and a 248-fold increase at 108 h of HP intake. However, among genes involved in the regulation of post-translational modifications and synthesis of intact FGF23 (*GALNT3*, *FAM20c*, and *FURIN*), only *FAM20c* expression showed a modest 1.7-fold increase at 12 h.

**Figure 5:**
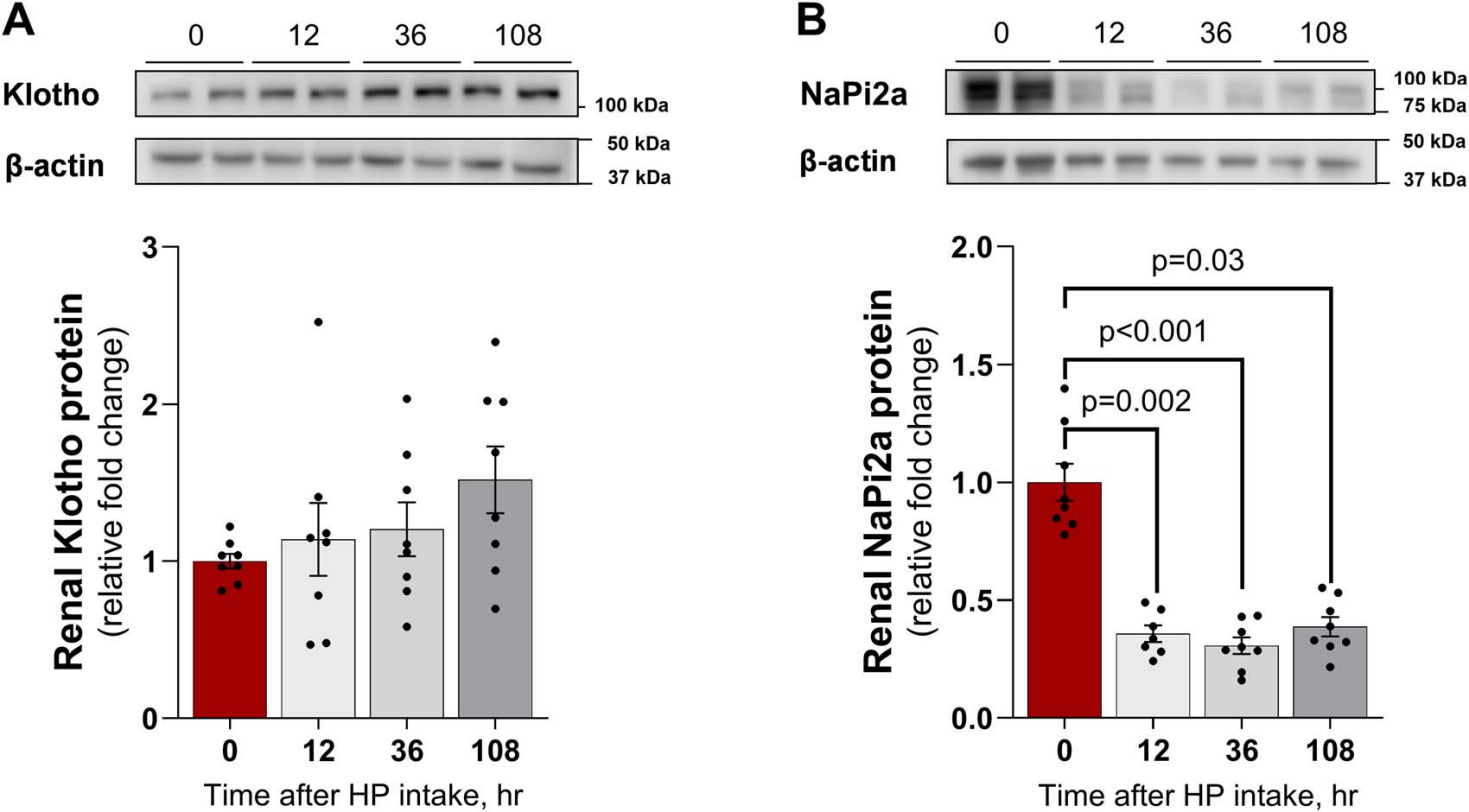
Renal protein expression of (A) αKlotho and (B) NaPi2a after 0, 12, 36, or 108 h of high phosphorus (HP) intake. Densitometry measurements were normalized to β-actin. Fold change in protein expression relative to 0 h. Data are presented as mean *+* SEM, *n* = 7-8 pigs per group. A *p*-value of <.05 was considered significant compared with time 0.

In the kidneys (Figure 4B), mRNA expression of the FGF23 receptor *FGFR1* (1.5-fold at 36 and 108 h) and co-receptor α*KLOTHO* (1.3-fold at 12 h) increased slightly. However, αKlotho protein levels were not affected by dietary P load (Figure 5A). The mRNA expression of 2 major renal sodium-dependent phosphate cotransporters, *NaPi2a* and *NaPi2b*, was significantly downregulated with the HP diet, with a 25% reduction at 108 h compared to baseline (Figure 4B). Consistent with these findings, Western blot analysis revealed decreased NaPi2a protein levels in the renal BBM from 12 h of HP feeding (Figure 5B).

In line with serum vitamin D metabolites findings, renal *CYP24A1* mRNA expression remained unchanged, whereas *Cyp27b1* was downregulated by 76% by 108 h of HP feeding (Figure 4B). Although significant changes were not observed in bone *Cyp27b1* and *CYP24A1* mRNA expression (Figure 4A), their expression patterns in response to P load were opposite to patterns expressed in the kidneys, suggesting local effects of dietary P intake on vitamin D metabolism.

## Discussion

The present study was designed to validate the roles of FGF23 in P homeostasis in juvenile, domestic swine. The current understanding of FGF23 synthesis and secretion, and roles in P homeostasis and vitamin D metabolism is largely derived from research involving FGF23-related renal pathological conditions in humans, and studies using genetically engineered murine models. The current results confirmed that these regulatory pathways were activated to restore P homeostasis when marginally deficient P diets were acutely replaced by diets with excess P.

Both domestic and miniature pigs are used as biomedical models, with justifications and advantages dependent upon unique constraints for specific experiments.^15–17^^,^^20^^,^^22^ The use of domestic swine for food production has provided an impetus to identify nutrient requirements to ensure the economic production of food with minimal inputs of limited resources, such as P. In contrast to food ingredients consumed in human diets that are marginal or limited in Ca, typical diets fed to domestic swine provide minimal amounts of P, due to basal feed ingredients and the relatively low costs of supplemental Ca and high costs of P.^18^ Thus, extensive research efforts by swine nutritionists have resulted in the development of deterministic models to predict Ca and P requirements^27^ with approaches that include the physiological importance of maintaining the Ca:P ratio in diets for domestic swine.^28^ Experiments designed with well established, defined requirements provide confidence for validation of expected outcomes. In this experiment, domestic, juvenile swine (barrows) were chosen based on the extensive database to support dietary requirements of P, Ca, and vitamin D.^19^^,^^27^^,^^28^

In the current experiment, pigs were fed a P-deficient diet followed by an acute transition to a high P diet to maximize homeostatic responses to dietary P intake. This approach was previously applied in studies with humans^29^ and mice^30^ to elucidate the temporal sequence for FGF23 regulation of P and vitamin D homeostasis. The acute transition in diets resulted in an initial rapid increase in both plasma and urinary P. The sustained elevation in urinary P excretion subsequently restored plasma P concentrations to the normal range. These findings, together with our previous studies in pigs,^25^ provide evidence for the immediate pathways that drive renal physiological adaptations to maintain P homeostasis in response to dietary P loads.

In human and murine models, FGF23 is well-established as a hormone derived from bone.^3^^,^^29^^,^^31^^,^^32^ Similarly, in pigs, gene expression of *FGF2*3 is predominantly localized to skeletal tissue.^33^ Consistent with the physiological roles of FGF23 in maintaining P homeostasis, the present study showed that *FGF23* mRNA expression in femoral cortical bone is rapidly upregulated in response to dietary P intake. These findings align with our previous study in sows,^23^ but contrast with another study with growing pigs that reported no changes in skeletal *FGF23* gene expression with high dietary P supply.^33^ This discrepancy may reflect differences in the timing of dietary treatments and physiological adaptations, as in our experiment we assessed acute responses to dietary changes, whereas the other study evaluated long-term adaptations to exposure. Inconsistencies in the regulation of *Fgf23* mRNA expression by dietary P intake have been previously reported in mice. Studies have shown that an increase in bone *Fgf23* expression is influenced by both dietary P intake and changes in serum P concentrations.^34^^,^^35^ In contrast, another study suggested that FGF23 production was regulated post-translationally, rather than at the gene expression level, in mice fed a high-P diet.^8^

The production of intact FGF23 in bone cells is regulated at post-transcriptional level through proteolytic cleavage. O-glycosylation of FGF23, mediated by the polypeptide N-acetylgalactosaminyltransferase 3 encoded by the *GALNT3* gene, protects FGF23 from proteolytic cleavage.^7^ In contrast, FAM20c-mediated phosphorylation of FGF23 inhibits its O-glycosylation, thereby promoting cleavage by the protease Furin.^9^ The importance of these post-transcriptional modifications in regulating intact FGF23 production was evident in humans with mutations in GALNT3 and FAM20C genes, which led to dysregulated levels of FGF23, and pathophysiological conditions associated with P metabolism.^36^^,^^37^ Under normal conditions, a high P diet fed to mice induced bone expression of *Galnt3* and circulating levels of full-length, intact FGF23, whereas changes in the expression of *Fam20c* and *Furin* were marginal in response to P load.^8^ The authors proposed that regulation of the *Galnt3* gene may serve as a mechanism for sensing extracellular P and regulation in the secretion of intact FGF23 to maintain P homeostasis. However, in the present study, a high P diet did not induce changes in bone mRNA expression of *GALNT3* and *FURIN*, while *FAM20c* showed a slight initial increase. Further evidence from healthy individuals and animal models is needed to better understand the regulation of gene expression and post-translational modifications that drive the synthesis and secretion of intact FGF23 under P load. Nonetheless, despite the need for further exploration of these mechanisms, the observation that high P intake increases circulating levels of FGF23 under physiological conditions have been widely reported.^31^^,^^35^^,^^38–40^

FGF23 exerts phosphaturic responses by action mediated through the FGFR1 receptor and the αKlotho co-receptor. The activation of this FGF23-mediated signaling pathway suppresses renal mRNA expression of type II sodium-dependent phosphate cotransporters, leading to increased urinary P excretion.^12^ The current results confirm this response in swine, as renal mRNA expression of *NaPi2a* and *NaPi2c* decreased with higher dietary P concentrations, which was accompanied by increased urinary P excretion. Additionally, protein abundance of NaPi2a in renal brush border membrane was downregulated, consistent with the FGF23-induced internalization and degradation of this sodium-phosphate cotransporter.^41^

Although the increase in dietary P was accompanied by a concurrent increase in dietary Ca, plasma Ca concentrations decreased following the acute dietary transition. Specifically, the dietary change elicited a rapid increase in plasma P concentrations and a concomitant decrease in plasma Ca concentrations. This inverse relationship between plasma Ca and P when concentrations of one of these minerals deviates from its physiological range was previously observed in swine^25^^,^^42^ and rat models.^43^ The increase in P intake initially resulted in higher plasma concentrations of PTH, the primary hormone involved in Ca homeostasis. The observed reduction in plasma Ca after switching to a HP diet may have triggered PTH secretion as the body attempted to restore normal Ca levels. Similar findings were reported in rats that received high concentrations of P either intravenously or orally.^43^ In this study, the increase in PTH also coincided with an increase in P excretion in urine. Beyond its role in Ca homeostasis, PTH acts as a phosphaturic hormone to reduce the expression of type II sodium-dependent phosphate cotransporters and to induce their internalization from the renal brush border membrane,^44^ a mechanism similar to that of FGF23. The initial increase in urinary P excretion may therefore be attributed to PTH action. However, after 26 h of high P intake, PTH levels declined while urinary P excretion continued to rise, indicating that other factors contribute to P excretion. Supporting evidence from studies in humans and mice indicates that FGF23 regulates urinary P excretion independently of PTH.^29^ Consistent with observations in this study, conclusions from human studies suggest that the initial elevation of PTH is critical for the rapid response to acute P loading, where later adaptations involve FGF23.^31^^,^^45^

Studies in mice have shown that FGF23 serves as a physiological regulator of vitamin D metabolism by reducing renal expression of 1-α-hydroxylase (*Cyp27b1*) and stimulating 24-hydroxylase (*Cyp24a1*), leading to a reduction in circulating concentrations of 1,25(OH)_2_D_3_.^46^ Consistently, the HP diet in this study downregulated renal *CYP27B1* and decreased plasma 1,25(OH)_2_D_3_. On the other hand, a previous study with sows showed that low P diets increased renal expression of *Cyp27b1*.^47^ This reduction in active vitamin D decreases intestinal P absorption, complementing renal adaptations to maintain P homeostasis under excessive dietary loads.

A limitation of this study was the inability to measure circulating levels of FGF23. Commercial ELISA kits are available to detect FGF23 in human samples and different animal species. However, available porcine FGF23 ELISA kits have not been extensively validated, raising concerns regarding the reliability of these porcine assays. As numerous studies investigating circulating FGF23 have been conducted in humans and mice, validated kits are readily available for these species. Despite the 77% protein homology between human and pig FGF23, and 76% homology between mouse and pig, we were unable to successfully measure FGF23 in pig samples using kits designed for human and mouse specimens. Although some manufacturers state that their kits may detect porcine FGF23 based on the epitope amino acid sequence homology used in assay development, these kits have not been validated using porcine biological matrices. We tested several kits according to recommended laboratory practices for ELISA validation.^48^ However, the results did not support reliable detection of FGF23 in porcine samples. Further inferences for physiological differences between human and porcine FGF23 proteins were recently identified in a pig-to-human kidney xenotransplantation study.^49^ Potential incompatibilities between circulating human FGF23 and porcine FGF23 renal receptors were inferred. The human recipient with a transplanted porcine kidney maintained elevated plasma intact FGF23 levels with hypocalcemia and hyperphosphatemia with markedly limited urinary excretion of P. These results infer that the porcine kidney receptors were possibly resistant to human FGF23.

## Conclusions

The data presented support the established roles of FGF23 as a phosphotropic hormone in the healthy porcine model. Pigs fed excess P upregulated bone *FGF23* mRNA expression. Consistent with the established roles of FGF23 for maintenance of P homeostasis, pigs exhibited reduced mRNA and protein abundance of renal sodium-dependent phosphate co-transporters, resulting in increased urinary P excretion and the restoration of normal plasma P concentrations. This phosphaturic effect occurred independently of circulating levels of PTH. Additionally, HP diets induced a decrease in renal *CYP27B1* mRNA expression and circulating levels of 1,25(OH)_2_D_3_ consistent with the role of FGF23 in regulation of vitamin D metabolism.

## Supplementary Material

SUPPLEMENTARY_MATERIAL_ziag129

## Data Availability

All reasonable requests for review of datasets can be made through contacting the corresponding author following publication.
