# Supplementary material for: Bone FGF23 expression and renal regulatory responses to excess phosphate consumption by juvenile swine
Source: JBMR Plus. 2026 Aug 8;10(10):ziag129. doi: 10.1093/jbmrpl/ziag129 (PMC13574284; doi:10.1093/jbmrpl/ziag129)
Supplement: Supplementary file 1 [file supplementary_material_ziag129.docx]

**SUPPLEMENTARY MATERIAL**

**Table S1.** Sequences of primers used in RT-qPCR

| Genes | Accession number |  | Primer sequence (5' to 3') |
| --- | --- | --- | --- |
| Target genes |  |  |  |
| *FGF23* | XM_001926525.4 | F | CAGCTACCACCTGCAGATCC |
|  |  | R | CCCTTAAGTCCATGCAGAGG |
| *GALNT3* | XM_021075049.1 | F | TTGGCCTTTGTGTAAAACCAG |
|  |  | R | TGCTTCAAGTCAAGTGGATTTC |
| *FURIN* | XM_021098918.1 | F | TGTGCCTTCCTGTGTGTAGC |
|  |  | R | AAGGCCCAGTCGTTAAATCC |
| *FAM20C* | XM_021085922.1 | F | GAAGGAACGGTCCCGAGATG |
|  |  | R | CCCTCTAGTCTTGGCTTGGC |
| *SOST* | XM_003131361.3 | F | TCAAGAACGATGCCACGGAA |
|  |  | R | AGGCGTCTTTGGTCTCGAAG |
| *FGFR1* | XM_005671767.3 | F | GCGACACCACCTACTTCTCC |
|  |  | R | CTCTGGGGATGTCCAGTACG |
| *KL* | XM_021065566.1 | F | GCTACAGCATCAGACGTGGA |
|  |  | R | TCCCTTCTAGGGGCTGATTT |
| *CYP27B1* | XM_021091124.1 | F | GAAAACCATGGTCCATTTGC |
|  |  | R | TCCGACTAGGAACCATGAGG |
| *CYP24A1* | NM_214075.2 | F | GCAAGAATGAAGCTTTTGGC |
|  |  | R | AGACACCAAGGTCAACCAGG |
| *SLC34A1* | NM_001044623.1 | F | CTTGTGGTAGCCTCCTTCAAC |
|  |  | R | GTGGCAATGCTGGTAATCAC |
| *SLC34A3* | XM_021081180.1 | F | GCACGCACCAGACATCCTC |
|  |  | R | GTGCCGCACCATCTCTTAATG |
| Housekeeping genes | |  |  |
| *RPS15* | NM_214334.1 | F | GCCAGAGGTAGTGAAGACGC |
|  |  | R | CACAGGCTTGTACGTGATGG |
| *RPL13A* | NM_001244068.1 | F | TGAGGTTGGCTGGAAGTACC |
|  |  | R | TCTCTACGTTCTTTTCCGCC |
| *ACTB* | XM_021086047.1 | F | CACGCCATCCTGCGTCTGGA |
|  |  | R | AGCACCGTGTTGGCGTAGAG |

*FGF23*, fibroblast growth factor 23; *GALNT3*, polypeptide N-acetylgalactosaminyltransferase 3; *FURIN*, furin; *FAM20C*, Golgi associated secretory pathway kinase; *SOST*, sclerostin; *FGFR1*, fibroblast growth factor receptor 1; *KL*, klotho; *CYP27B1*, 25-hydroxyvitamin D3 1alpha-hydroxylase; *CYP24A1*, cytochrome P450 family 24 subfamily A member 1; *SLC34A1*, solute carrier family 34 member 1; *SLC34A3*, solute carrier family 34 member 3; *RPS15*, ribosomal protein S15; *RPL13A*, ribosomal protein L13a; *ACTB*, actin beta
